# Compared analysis of physiology and transcriptomics reveals superior cold tolerance in CV-1 compared to K326

**DOI:** 10.3389/fpls.2025.1704700

**Published:** 2025-11-26

**Authors:** Quanliu Yang, Caixian Wang, Ting Luo, Dandan Yang, Jianyu Zhu, Min Gan, Yuange Yu

**Affiliations:** 1 Guizhou Academy of Tobacco Sciences, Guiyang, China; 2 School of Minerals Processing and Bioengineering, Central South University, Changsha, China

**Keywords:** physiology, transcriptomics, cold-treatment, tobacco, weighted gene co-expression network analysis

## Abstract

**Introduction:**

Low-temperature stress can cause damage to the growth and development of tobacco plants and the yield and quality of tobacco leaves.

**Methods:**

To elucidate the physiological and molecular mechanisms underlying the differing responses of different tobacco varieties to low-temperature stress at 6 °C, transcriptomics analysis was employed to investigate the differences in physiological and gene regulatory networks between K326 and CV-1.

**Results:**

Morphological analysis revealed that CV-1 recovered more quickly to its pre-cold stress state than K326 during the later stages of low-temperature stress, demonstrating stronger cold tolerance. Physiological and biochemical analyses showed that compared to K326, CV-1 exhibited stronger antioxidant enzyme activities (superoxide dismutase, catalase, peroxidase) and lower membrane lipid peroxidation damage, as indicated by the decreased malondialdehyde content. Differential gene expression analysis indicated that the enhanced cold tolerance of CV-1 may be attributed to stronger phenylalanine synthesis capacity, NADH synthesis, and antioxidant enzyme activities. Weighted co-expression network analysis revealed that the enhanced cold tolerance of CV-1 may be attributed to its unique SUMOylation and phosphorylation regulatory pathways of proteins such as *DeSI1L*, *EDS1L*, and *EXPA2L*. Compared to K326 under low-temperature stress, CV-1 also exhibits stronger photosynthetic capacity, hydrogen peroxide transport capacity, and isoflavone synthesis capacity. Additionally, CV-1 shows higher expression of the SPFH protein superfamily and heat shock protein family.

**Discussion:**

This study revealed differences in gene regulatory networks between K326 and CV-1 in response to low-temperature stress and identified candidate genes associated with low-temperature stress, which can be utilized for genetic improvement of tobacco plants to enhance their cold tolerance.

## 1 Introduction

Tobacco (*Nicotiana tabacum* L.), as a globally cultivated important cash crop, is a warm-loving plant that is highly sensitive to low-temperature stress (Ma et al., 2020; Li et al., 2023b). When temperatures drop too low, tobacco’s photosynthesis weakens, severely affecting leaf yield and quality, resulting in a significant decline in economic benefits (Ding et al., 2019; He et al., 2020). Tobacco is native to tropical and subtropical regions and, through long-term evolutionary adaptation, has not developed a complete low-temperature response mechanism (Suo et al., 2024). When faced with low-temperature stress in the environment, it is prone to cellular damage, metabolic disorders, and physiological dysfunction (Kidokoro et al., 2022; Xie et al., 2025). K326 and CV-1 are widely cultivated tobacco varieties in China. CV-1 was discovered in 2006 in a tobacco field in Chenzhou, Hunan Province, China. It is an excellent cultivar developed by Guizhou Academy of Tobacco Sciences and was bred from a naturally occurring field mutation of Yunyan 87. Yunyan 87 itself resulted from crossing K326 as the male parent with Yunyan 2 as the female parent. CV-1 exhibits a cylindrical plant structure. This strain naturally develops approximately 28 leaves and reaches a natural plant height of 165 cm. After topping, it retains about 22 leaves and maintains a plant height of approximately 122 cm. The impact of low temperature on their economic benefits is extremely severe. Cold stress inhibits the synthesis of phenolic compounds in tobacco cells, which are key components of plant stress resistance. Additionally, cold temperatures disrupt the steady state of antioxidant enzyme defense mechanisms dominated by superoxide dismutase (SOD), peroxidase (POD), and catalase (CAT), leading to the accumulation of membrane-derived peroxidation products, loss of membrane integrity, electrolyte leakage, and elevated malondialdehyde (MDA) levels (Gu et al., 2024). Although key response pathways for plants to cold stress have been identified in model plants such as Arabidopsis, such as the ICE-CBF-COR regulatory pathway, the molecular mechanisms underlying the differential responses of different tobacco varieties to cold stress remain unexplained (Li et al., 2013; Gusain et al., 2023).

The emergence of high-throughput sequencing technology has greatly promoted the widespread application of transcriptomics research in the field of plant science. Such research has covered many plant species, including model crops such as Arabidopsis, food crops such as corn and rice, and tobacco (Montes et al., 2022; Xiao et al., 2024; Wei et al., 2025). These studies have provided important insights into the transcriptional regulatory mechanisms involved in plant responses to cold stress. Furthermore, recent multi-omics studies on tobacco have shown that cold tolerance involves coordinated changes in gene expression related to hormone signaling, antioxidant defense, and secondary metabolism. Compared to cold-sensitive varieties like CB-1, cold-tolerant varieties like K326 exhibit upregulation of *DREB* genes, enhanced accumulation of osmoprotective substances like sugars and amino acids, and reduced levels of the hormone signaling molecule ethylene (Jin et al., 2017). Another multi-time point comparative transcriptomics analysis of Tai tobacco (cold-sensitive type) and Yun tobacco (cold-tolerant type) identified differential co-expression networks between the two, pinpointing hub genes such as *ABI3/VP1*, *ARR-B*, and *WRKY* transcription factors that regulate the differences in cold tolerance between different tobacco varieties (Luo et al., 2022). Additionally, transgenic tobacco plants overexpressing the *ICE1* gene exhibited improved photosynthetic efficiency and reduced malondialdehyde levels under low-temperature stress, further confirming the critical role of key hub genes in responding to low-temperature stress (Zhang et al., 2018). Although some progress has been made, systematic comparisons of transcriptional networks between cold-tolerant and cold-sensitive tobacco varieties remain insufficient, particularly regarding the dynamic expression patterns of key genes during cold stress responses.

Therefore, it is necessary to study and compare the cold stress responses of multiple tobacco varieties to investigate differences in their cold stress response mechanisms. In this study, comparing the phenotypic, physiological, biochemical, and gene expression differences between K326 and CV-1 helps reveal distinct cold stress response pathways in different tobacco varieties. This has profound implications for the breeding and improvement of cold-tolerant tobacco cultivars.

## 2 Materials and methods

### 2.1 Tobacco cultivation and low-temperature treatment

Two distinct cultivars of tobacco (*Nicotiana tabacum* L.), K326 and CV-1, were cultivated. Seeds were obtained from the Guizhou Tobacco Science Research Institute, China. Plants were grown in an artificial climate chamber under controlled conditions: 70% relative humidity, a 16-hour light/8-hour dark photoperiod, and a constant temperature of 25 °C. Sixty-day-old seedlings were subjected to low-temperature treatment at 6 °C. Leaf samples from both control (untreated) and cold-treated plants were collected at 0, 24, 48, and 96 hours after treatment initiation. All collected samples were immediately flash-frozen in liquid nitrogen and stored at -80 °C for subsequent analysis. All results in this study are based on the average of three independent biological replicates.

### 2.2 Physiological and biochemical parameter quantification

Tobacco leaves used for physiological and biochemical experiments weighed 0.1 g. The activities of POD, SOD, and CAT, along with the contents of MDA were utilized to evaluate the degree of low-temperature stress in tobacco cells. All physiological and biochemical parameters were determined using commercial assay kits (Solarbio, Beijing, China; BC0095, BC0175, BC0205, BC0025).

### 2.3 Transcriptome sequencing and data analysis

Total RNA was extracted from fresh tobacco leaves. The integrity and quantity of RNA were assessed using the Agilent 2100 Bioanalyzer. Polyadenylated mRNA was enriched from total purified RNA using Oligo (dT) magnetic beads. Sequencing libraries were prepared with the Illumina TruSeq RNA Sample Preparation Kit following the manufacturer’s protocol. All libraries underwent paired-end sequencing on the Illumina platform. Raw sequencing data underwent stringent quality control and filtering to ensure data reliability.

The reference genome and corresponding gene annotation files were downloaded from the designated genome database. The reference genome index was constructed using HISAT2 v2.0.5, and paired-end clean reads were aligned to the reference genome with the same software. *De novo* transcript assembly and novel gene prediction were performed using StringTie (v1.3.3b), which employs a network flow algorithm combined with optional ab initio assembly to reconstruct transcripts. Gene expression quantification was conducted using featureCounts (v1.5.0-p3) to calculate read counts mapped to each gene. Transcript abundance was normalized as FPKM (Fragments Per Kilobase of transcript per Million mapped reads) based on gene length and mapped read counts.

### 2.4 Differential gene expression analysis

Differential expression analysis between two comparative groups was performed using the DESeq2 package (version 1.20.0) in R (version 3.0.3). Raw read counts were normalized and modeled using the negative binomial distribution to identify differentially expressed genes (DEGs). Genes with |log2(fold change)|>1 and adjusted p-value (padj) < 0.05 were defined as statistically significant DEGs.

For clustering analysis of DEGs, hierarchical clustering was visualized using the ggplot2 and pheatmap packages. Venn diagrams generated by the VennDiagram package were employed to illustrate the overlap of DEGs across different tobacco treatment groups (Anders and Huber, 2010).

Gene Ontology (GO) and Kyoto Encyclopedia of Genes and Genomes (KEGG) enrichment analyses were conducted using the clusterProfiler package (version 3.8.1). To mitigate gene length bias, the analysis incorporated background correction based on the entire genome. Enriched GO terms and KEGG pathways with a Benjamini-Hochberg-adjusted p-value < 0.05 were considered statistically significant.

### 2.5 Co-expression networks analysis

The gene co-expression networks of two tobacco varieties were analyzed using the WGCNA package in R (Version 3.5.0) to calculate weighted association-based functional clusters. A one-step network construction and module detection approach was employed to generate co-expressed gene networks, followed by the identification of functional modules (Langfelder and Horvath, 2008). Eigengenes were utilized to represent inter-module associations and intra-variety correlations at each time point. All co-expressed genes were clustered into 21 distinct modules corresponding to specific varieties and time points. Gene networks within each module were visualized using Cytoscape (v3.9.0).

### 2.6 QRT-PCR assay

The extracted total RNA was utilized for quantitative reverse transcription polymerase chain reaction (qRT-PCR) analysis. Following verification of RNA purity meeting required standards, the RNA was reverse-transcribed into complementary DNA (cDNA). Subsequent analytical processing involved normalization using *Ntubc2* as the reference gene, with relative expression levels calculated through the 2^-ΔΔCt^ method. The primers used for qRT-PCR are listed in **Supplementary Table S1**.

## 3 Results

### 3.1 Determination of phenotypic characteristics and physiological and biochemical parameters before and after low-temperature treatment

**Figure 1:**
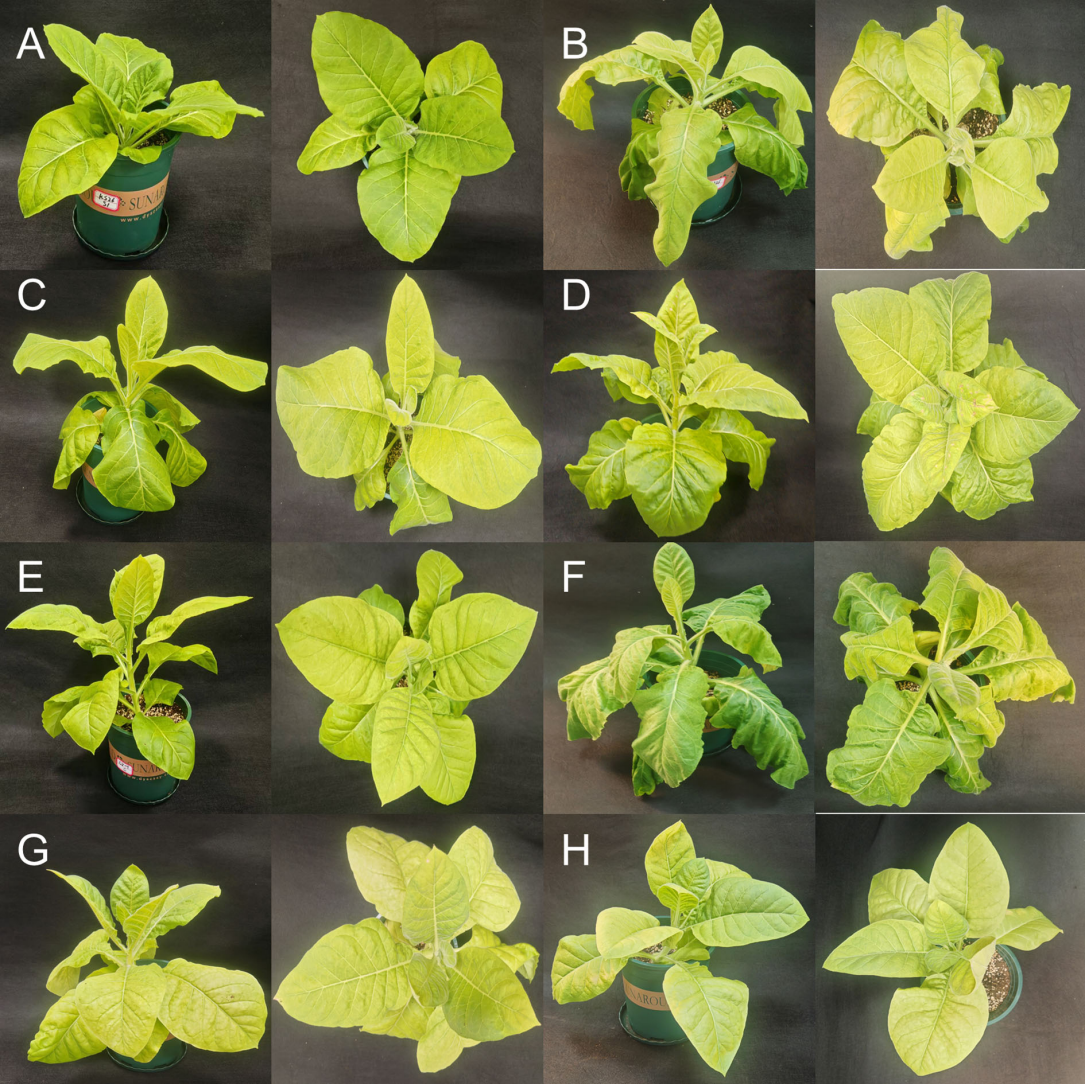
Phenotypic changes of two tobacco varieties (K326 and CV-1) before and after low-temperature treatment. **(A)** K326 before cold treatment, **(B)** K326 after 1 day of cold treatment, **(C)** K326 after 2 days of cold treatment, **(D)** K326 after 4 days of cold treatment, **(E)** CV-1 before cold treatment, **(F)** CV-1 after 1 day of cold treatment, **(G)** CV-1 after 2 days of cold treatment, **(H)** CV-1 after 4 days of cold treatment.

When subjected to low-temperature treatment, both K326 and CV-1 tobacco cultivars exhibited similar phenotypic changes (**Figure 1**). In both cultivars, leaf wilting reached its maximum severity one day after treatment initiation, with all leaves except the top two or three showing pronounced wilting and wrinkling. At 24 h, K326 had two leaves completely wilted and three leaves marginally wilted, while CV-1 had four leaves completely wilted and three leaves marginally wilted. Starting from the second day of low-temperature exposure, the wilting symptoms progressively alleviated, and leaf gradually recovered. At 48 h, four leaves were marginally wilted in K326 and only two leaves were marginally wilted in CV-1. At 96 h, two leaves were marginally wilted in K326, and no leaves were visibly wilted in CV-1.

Comparative analysis revealed that K326 displayed less severe wilting than CV-1 after 24 hours of treatment, indicating initial stronger cold resistance in K326. However, the recovery dynamics differed significantly between the two cultivars: K326 exhibited slower recuperation from the second day onward. By the fourth day of treatment, CV-1 had largely restored its pre-treatment morphology, whereas K326 still showed incomplete recovery of wrinkling at the edges of basal leaves. This observation demonstrates that K326 possesses weaker adaptive capacity to prolonged low-temperature stress compared to CV-1.

**Figure 2:**
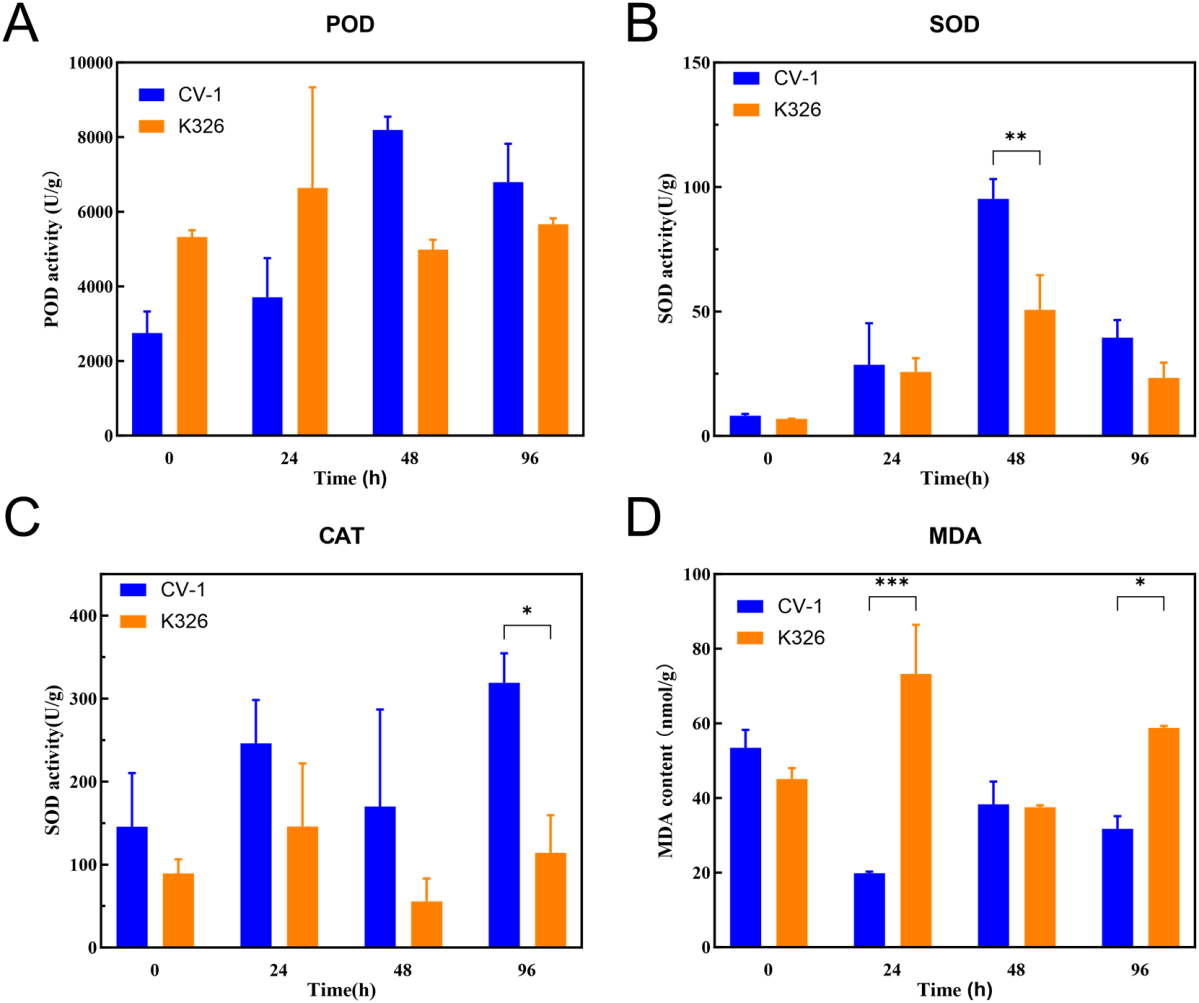
The changes in physiological activities of K326 and CV-1 before and after low-temperature treatment. **(A)** POD, **(B)** SOD, **(C)** CAT, **(D)** MDA. Means were compared by Šídák’s test. *, ** and *** indicate P < 0.05, P < 0.01 and P < 0.001.

The biochemical analysis revealed that both K326 and CV-1 cultivars exhibited an initial significant increase followed by a gradual decrease in antioxidant enzyme activities under low-temperature stress. The POD activity in CV-1 reached its maximum at 48 h of cold treatment (**Figure 2A**), showing a 23% higher peak activity than that observed in K326, indicating that CV-1 possesses a stronger capability to scavenge peroxides. Furthermore, CV-1 demonstrated a marked advantage in superoxide dismutase SOD activity at 48 h (**Figure 2B**), suggesting its enhanced efficiency in eliminating superoxide radicals during the mid-phase of cold stress. Simultaneously, the CAT activity of CV-1 remained consistently higher than that of K326 throughout the entire stress period (**Figure 2C**). In contrast, K326 exhibited higher MDA content than CV-1 during cold exposure (**Figure 2D**), reflecting more severe membrane lipid peroxidation damage in its cellular structure.

Collectively, CV-1 displayed significantly superior cold resistance compared to K326 through synergistic interactions of elevated antioxidant enzymes (POD/SOD/CAT) and reduced accumulation of membrane lipid peroxidation products. Transcriptome analysis also showed that antioxidant enzyme system-related genes were expressed at higher levels in CV-1 than in K326, such as catalase isozyme 1 (LOC107826360, LOC107828252), peroxidase 51&63 (LOC107804632, LOC107794292), superoxide dismutase (LOC107829594).This also validates at the molecular level that CV-1 is more cold-tolerant than K326.Notably, the antioxidant defense system and membrane protection mechanisms of CV-1 operated more efficiently during the mid-to-late stages of cold stress (48–96 h), which likely constitutes the key physiological basis for its enhanced cold tolerance. Conversely, K326 exhibited insufficient antioxidant capacity and heightened vulnerability of its membrane system, ultimately leading to weaker cold resistance.

### 3.2 Comparative analysis of differentially expressed genes between K326 and CV-1

**Figure 3:**
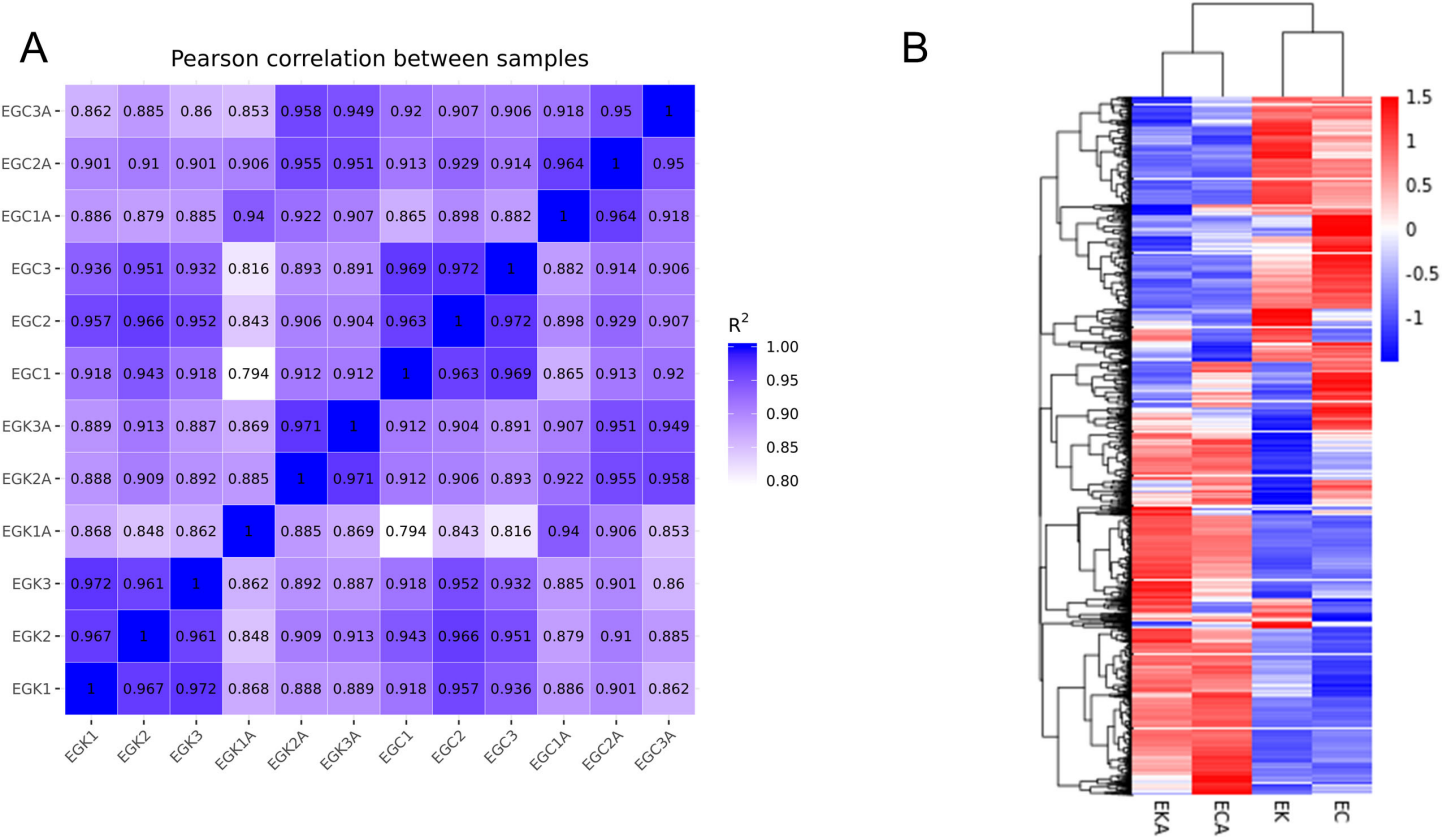
**(A)** Pearson correlation analysis of K326 and CV-1, **(B)** Heatmap of differentially expressed genes before and after low temperature treatment with K326 and CV-1.

To investigate the differences in cold hardness among tobacco cultivars, leaf tissues of K326 and CV-1 before and after low temperature treatment were selected for transcriptome sequencing. In the experiment, we designed four experimental groups, EK (experimental groups of K326 before low temperature treatment), EKA (experimental groups of K326 at 96 hours after low temperature treatment), EC (experimental groups of CV-1 before low temperature treatment), ECA (experimental groups of CV-1 at 96 hours after low temperature treatment). To test for correlations between sequenced samples, we performed Pearson’s correlation analysis of the data (**Figure 3A**). It can be observed that the correlation between the samples of each experimental group is high, and the EKA and ECA after low temperature treatment show significant differences compared with EK and EC before low temperature treatment.

In order to visualize the differences in gene expression between K326 and CV-1 before and after low temperature treatment, we plotted a cluster heat map for the differentially expressed genes of the two tobaccos (**Figure 3B**). Red represents the high expression of genes, blue represents the low expression of genes, and different differentially expressed genes are divided into different clustering regions. The different clustering regions of differentially expressed genes between K326 and CV-1 before and after low temperature treatment may be the reason for the difference in cold resistance between K326 and CV-1. Also, to quantify the number of differentially expressed genes in the two tobaccos, K326 and CV-1, before and after low temperature treatment, we plotted the volcano plot of the expressed genes in the two tobaccos before and after low temperature treatment.

**Figure 4:**
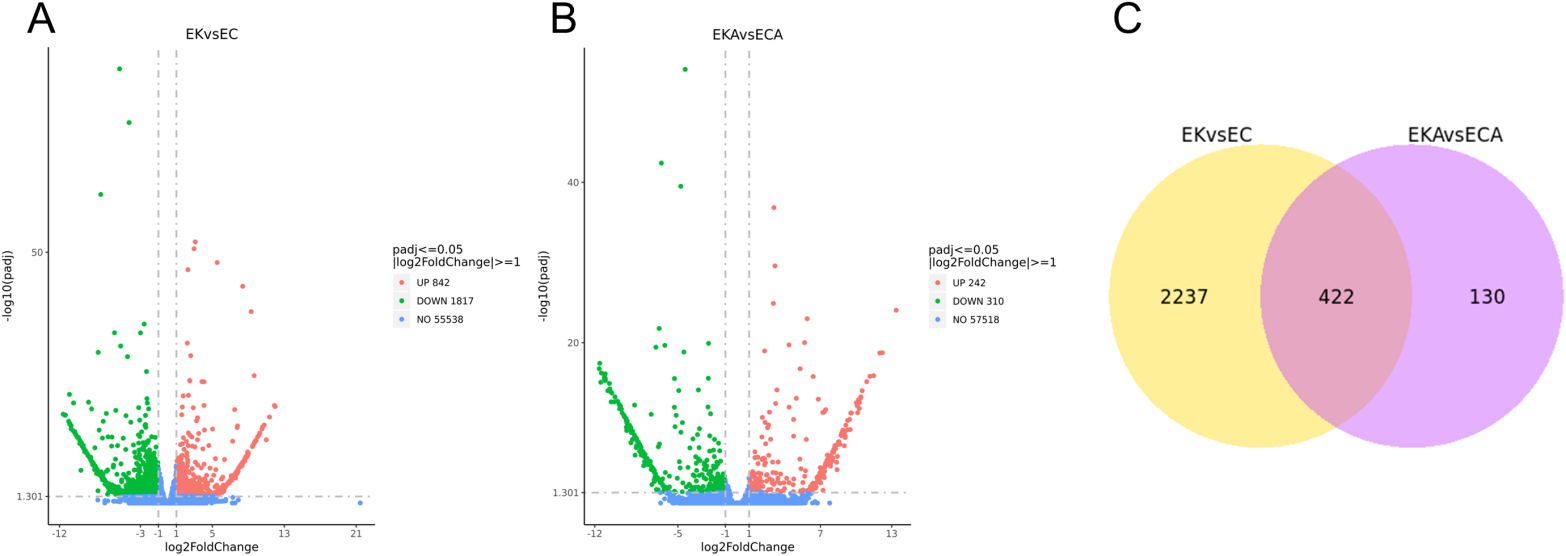
Comparison of volcano plots before **(A)** and after **(B)** low temperature treatment with K326 and CV-1, **(C)** Venn plots between the two comparison combinations.

A total of 2659 differentially expressed genes were identified between EK vs EC group, of which 842 genes were up-regulated and 1817 genes were down-regulated (**Figure 4A**). A total of 552 differentially expressed genes were identified between EKA vs ECA group, of which 242 genes were up-regulated and 310 genes were down-regulated (**Figure 4B**). Following low temperature treatment, the differential genes between K326 and CV-1 were significantly decreased, indicating that the adaptation to low temperature is similar in both tobacco species (**Table 1**). Venn diagrams between the two comparison combinations indicate that 2237 genes are unique to EK vs EC group, 130 genes are unique to EKA vs ECA group, and 422 genes are common to both comparison combinations (**Figure 4c**). These 422 genes may contribute to the differences in cold resistance between K326 and CV-1. Most of the CV-1 genes have higher gene expression levels than K326, and also better regulate physiological activities in response to cold stress, showing higher cold tolerance.

**Table 1:** Number of differentially expressed genes in different comparison groups.

Compare	Up	Down	All
EK vs EC group	842	1817	2659
EKA vs ECA group	552	242	310

In EKA vs ECA group, many gene families related to low temperature stress have also been identified, such as *bHLH* (basic helix-loop-helix), *NAC* (*NAM*, *ATAF1/2* and *CUC1/2*), *AP2/ERF* (*APETALA2*/ethylene-responsive factor), etc. Studies have shown that *bHLH* family genes in tobacco directly bind to the G-box/E-box motif in the promoter of *NtCBF* gene and positively regulate its expression, and plants overexpressing *bHLH* family genes show lower electrolyte leakage, malondialdehyde content, H_2_O_2_ and reactive oxygen species (ROS) accumulation reduction under cold stress (Zhao et al., 2018). The expression of *NAC* gene family was up-regulated under drought stress and abscisic acid (ABA), but down-regulated under cold stress (Huang et al., 2016). Overexpression of *AP2/ERF*, on the other hand, leads to accumulation of COR peptides, Pro, and soluble sugars in plant cells (Gilmour et al., 2000). Compared with K326, most of the *bHLH* (such as LOC107824933, LOC107799156, etc.), *NAC* (such as LOC107777238, LOC107764171, etc.), *AP2/ERF* (such as LOC107810152, LOC107830873, etc.) transcription factors in CV-1 were expressed at higher levels after low-temperature stress levels were all higher, resulting in the expression of higher antioxidant enzyme activities and MDA levels and greater cold tolerance.

### 3.3 GO enrichment and KEGG enrichment analysis of differentially expressed genes before and after low temperature treatment in K326 and CV-1

To elucidate how the differentially expressed genes contribute to the difference in cold resistance between K326 and CV-1 by affecting the physiological activities of cells, GO enrichment and KEGG enrichment analysis were performed on the differentially expressed genes before and after low temperature treatment.

**Figure 5:**
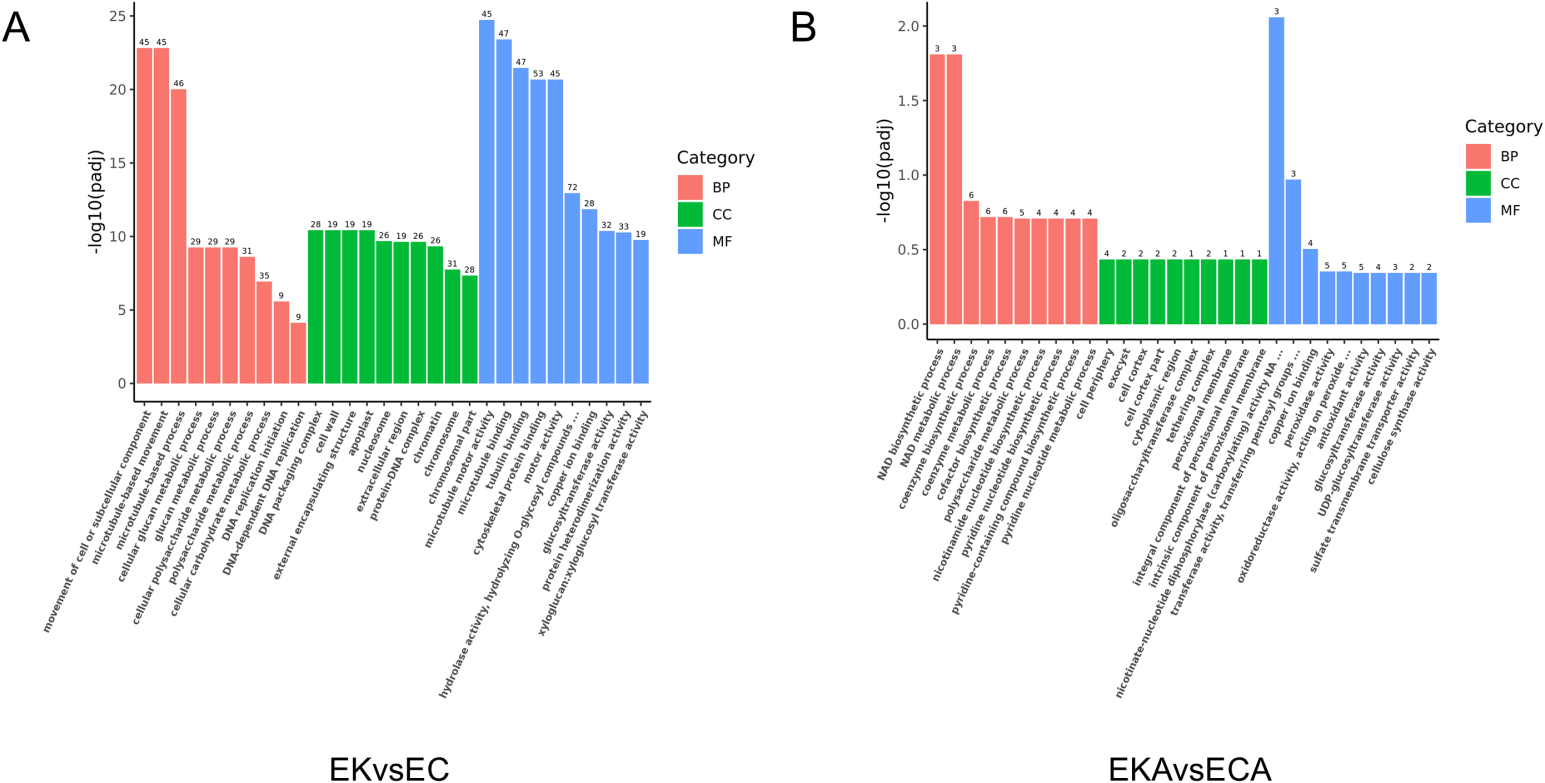
GO enrichment of differentially expressed genes. **(A)** before cold treatment, **(B)** after cold treatment.

Histograms were drawn based on the thirty significant terms of GO enrichment results and twenty significant terms of KEGG enrichment results. GO enrichment included three aspects: biological process (BP), cellular component (CC) and molecular function (MF). GO analysis of DEGs in EK vs EC group comparison combination showed that BP terms such as movement of cell or subcellular component, microtubule-based movement, microtubule-based process were significantly enriched. In the CC terms, such as cell wall, external encapsulating structure, apoplast were significantly enriched. In the MF terms, such as microtubule binding, tubulin binding, cytoskeletal protein binding were significantly enriched (**Figure 5A**). GO analysis of DEGs in EKA vs ECA group comparison combination showed that BP terms such as NAD biosynthetic process, NAD metabolic process, coenzyme biosynthetic process were significantly enriched. In the CC terms, such as cell periphery, exocyst, cell cortex were significantly enriched. In the MF terms, such as nicotinate−nucleotide diphosphorylase (carboxylating) activity, transferase activity, copper ion binding, peroxidase activity, oxidoreductase activity, acting on peroxide as acceptor, antioxidant activity, glucosyltransferase activity were significantly enriched (**Figure 5B**).

**Figure 6:**
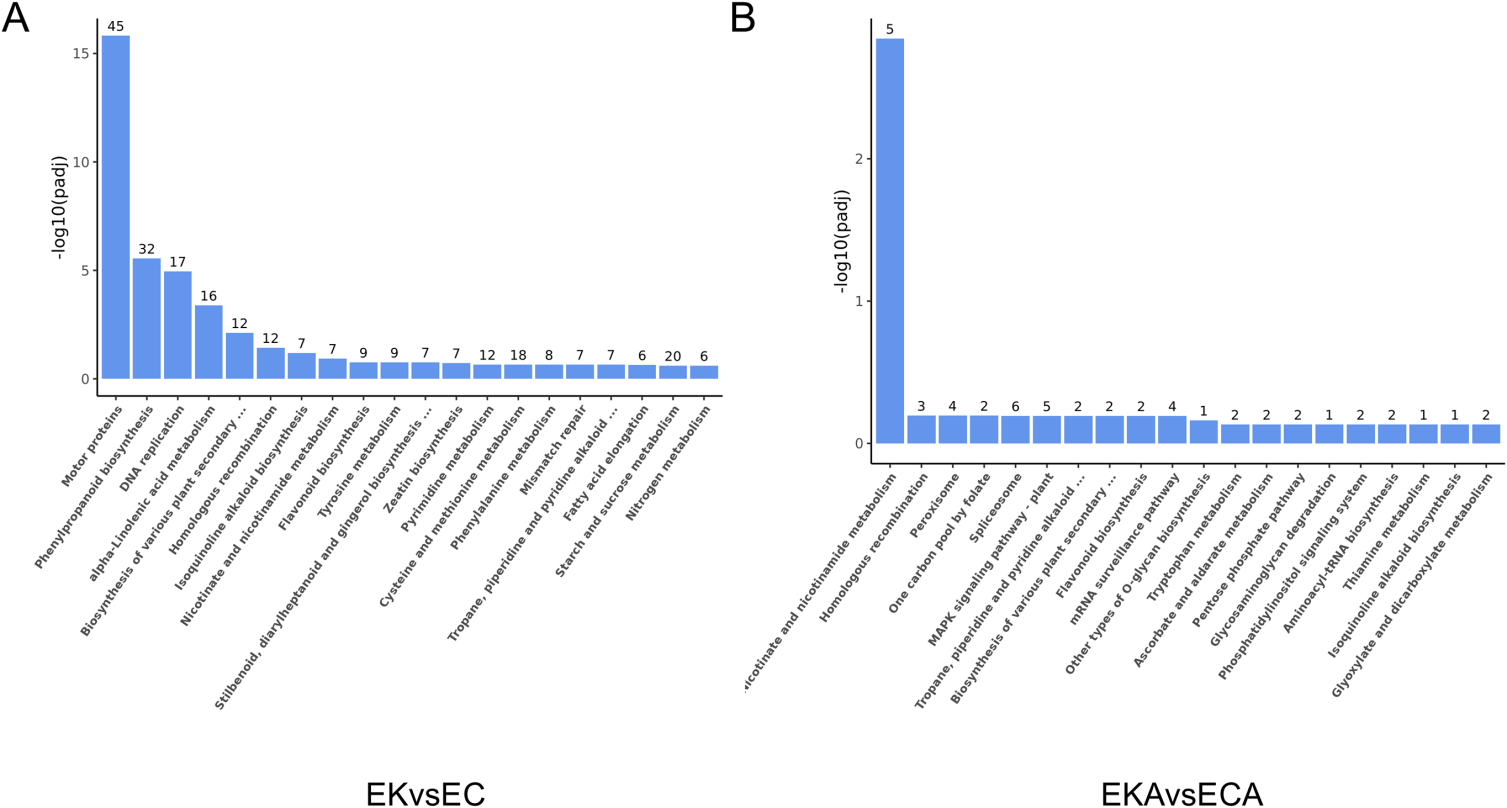
KEGG enrichment of differentially expressed genes. **(A)** before cold treatment, **(B)** after cold treatment.

KEGG enrichment analysis showed that EK vs EC group was enriched in motor proteins, phenylpropanoid biosynthesis, DNA replication, alpha−Linolenic acid metabolismand other pathways (**Figure 6A**). In EKA vs ECA group, nicotinate and nicotinamide metabolism, homologous recombination, peroxisome, one carbon pool by folateother pathways were enriched (**Figure 6B**).

Nicotinate can be used to synthesize coenzymes NADH and NADPH in plant cells, which can help to scrape free radicals and protect cells from oxidative stress damage (Wei et al., 2019). The increase of peroxidase activity and the increase of soluble sugar are also the manifestations of plant response to cold stress. Interestingly, copper ion binding was enriched in EKA vs ECA group, suggesting its possible role in plant coping with low temperature (Solier et al., 2023). Compared with K326, CV-1 showed higher expression levels of genes related to NADH synthesis capacity and antioxidant enzyme activities, thus synthesizing more NADH and antioxidant enzymes to protect it from damage by ROS, and exhibited higher cold tolerance.

### 3.4 Weighted gene co-expression network analysis before and after low temperature treatment in K326 and CV-1

Through weighted gene co-expression network analysis (WGCNA), genes with similar expression patterns in two tobacco varieties (K326 and CV-1) under low-temperature treatment and control conditions were clustered into modules to identify hub genes within these modules, thereby investigating the underlying mechanisms contributing to cold resistance differences between K326 and CV-1.

**Figure 7:**
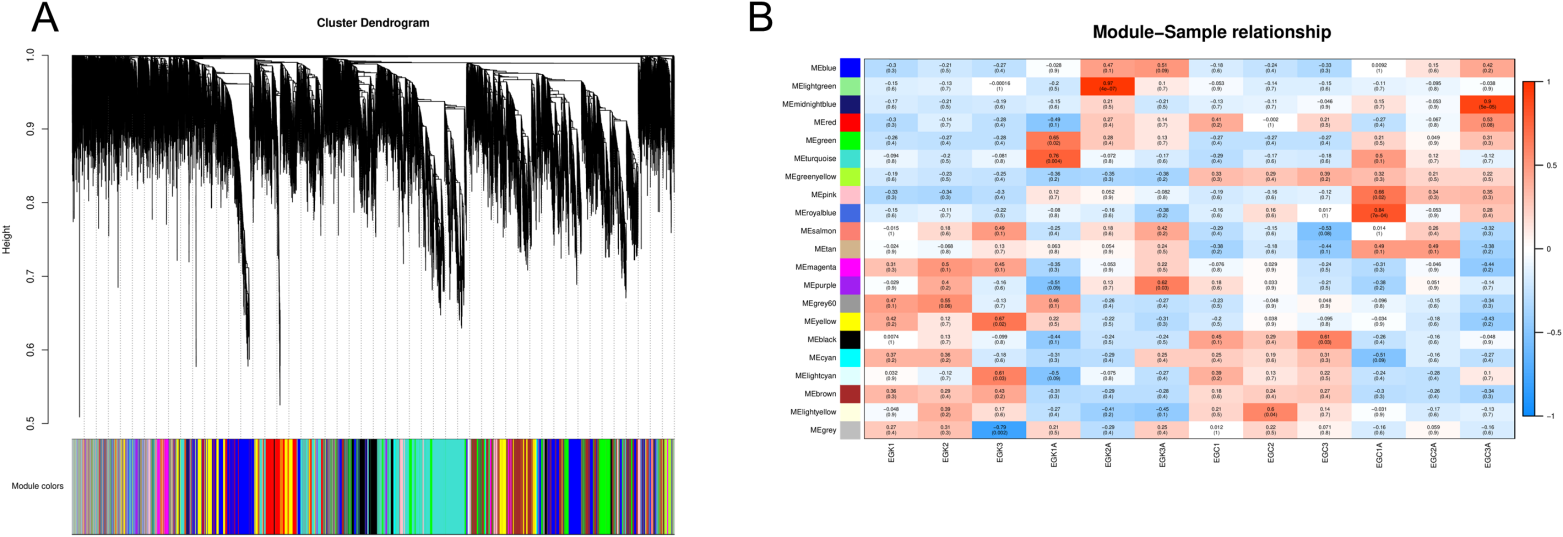
**(A)** Hierarchical modular clustering tree of K326 and CV-1 before and after low temperature treatment, **(B)** Module-sample relationship heat map.

After setting an appropriate Pearson correlation coefficient threshold, we constructed a cluster tree using the WGCNA R package to analyze gene expression profiles of K326 and CV-1 before and after low-temperature treatment, identifying 21 distinct modules represented by different colors (**Figure 7A**). The module-sample relationship heatmap revealed that the green module exhibited a negative correlation with both K326 and CV-1 under control conditions but switched to a positive correlation after low-temperature treatment (**Figure 7B**). In contrast, the brown module showed the opposite pattern: a positive correlation under control conditions and a negative correlation post-treatment. Notably, the pink module displayed a strong positive correlation with CV-1 after low-temperature treatment, while showing negative correlations with both varieties under control conditions. This suggests that genes within the pink module may be associated with enhanced cold resistance in CV-1.

**Figure 8:**
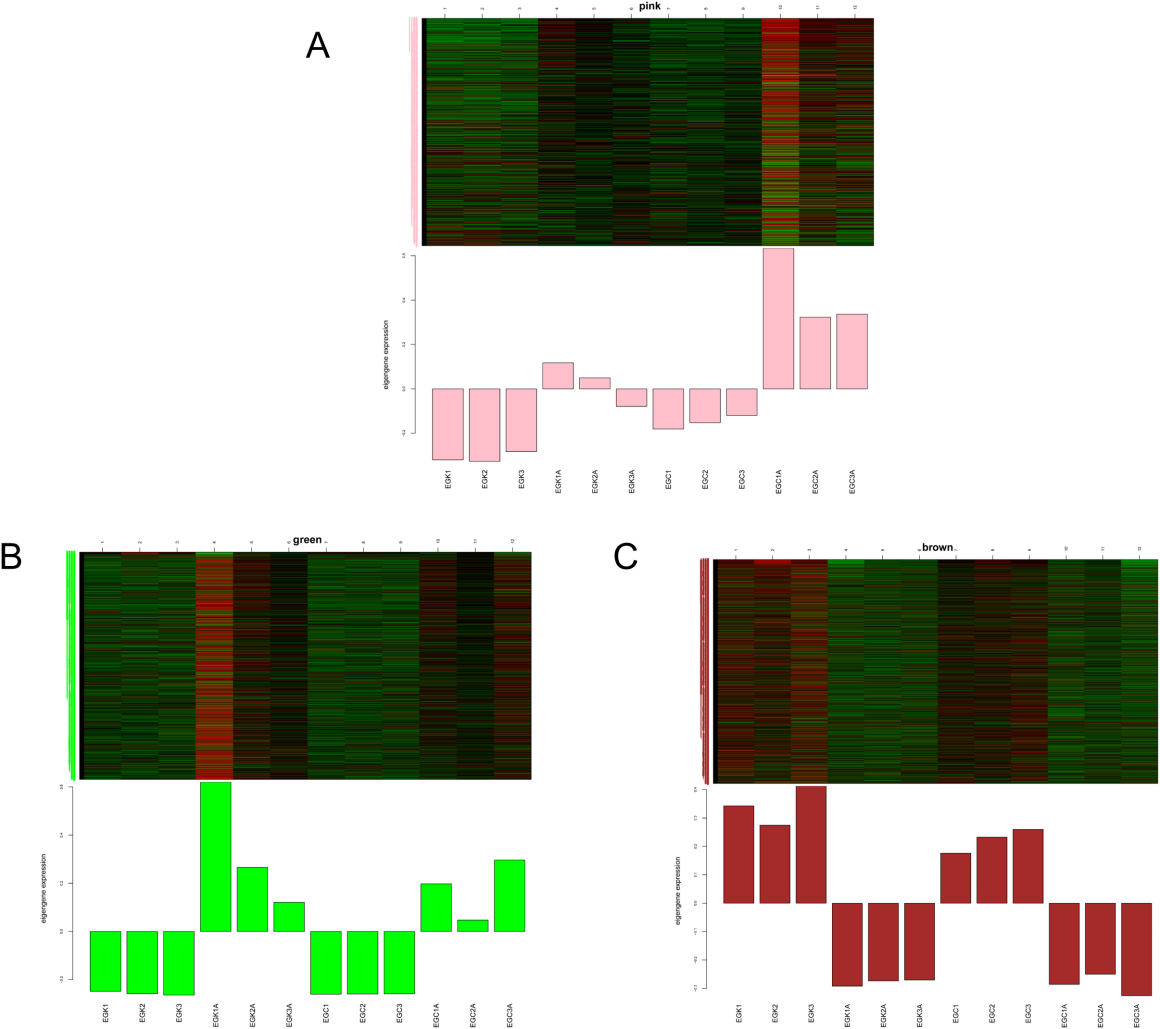
Expression patterns of genes in different modules. **(A)** pink module, **(B)** green module, **(C)** brown module.

We constructed a visual view of the changes in gene expression patterns in the three modules before and after low temperature treatment (**Figure 8**). Before low temperature treatment, the expression of characteristic genes in the pink module of K326 and CV-1 was weakened, but after low temperature treatment, the expression of characteristic genes in CV-1 was significantly higher than that in K326 (**Figure 8A**). Before low temperature treatment, the expression of characteristic genes in the green module of K326 and CV-1 was weakened, but after low temperature treatment, the expression of characteristic genes in K326 was increased compared with CV-1 (**Figure 8B**). The expression of signature genes in the brown module of K326 and CV-1 was enhanced before low temperature treatment, and the expression of signature genes in both of them was significantly decreased after low temperature treatment (**Figure 8C**).

The pink module contains 1172 genes, the brown module contains 4957 genes, and the green module contains 2826 genes. Important genes in each module were screened based on the connectivity of each gene with other genes. The k-Within value of each gene indicates its connectivity with other genes within the module (**Supplementary Table S2**). The top 10 genes were selected from each module as the hub genes for that module based on their k-Within ranking (**Table 2**).

**Table 2:** Top 10 hub genes in each module.

Gene_ID	Gene_name	Gene_description	Module	k-Within
107759334	LOC107759334	desumoylating isopeptidase 1-like	pink	373.6318956
107811391	LOC107811391	protein EDS1-like	pink	373.5170682
107826487	LOC107826487	methylthioribose kinase-like	pink	365.8521238
107784433	LOC107784433	expansin-like A2	pink	360.5444267
107831052	LOC107831052	calmodulin-binding protein 60 B-like	pink	358.014892
107814674	LOC107814674	mechanosensitive ion channel protein 10-like	pink	353.4439599
107823549	LOC107823549	nudix hydrolase 8-like	pink	349.8397961
107772124	LOC107772124	3-hydroxy-3-methylglutaryl-coenzyme A reductase 1	pink	348.1558339
107812736	LOC107812736	galactan beta-1,4-galactosyltransferase GALS3	pink	346.9346858
107775062	LOC107775062	protein NRT1/PTR FAMILY 2.11-like	pink	345.2019755
107824278	LOC107824278	pyruvate kinase isozyme A	brown	1442.376145
107832425	LOC107832425	ARM REPEAT PROTEIN INTERACTING WITH ABF2-like	brown	1438.979558
107818571	LOC107818571	probable ADP-ribosylation factor GTPase-activating protein AGD6	brown	1424.546142
107784193	LOC107784193	FAM10 family protein At4g22670-like	brown	1396.179403
107825853	LOC107825853	probable aquaporin PIP-type pTOM75	brown	1395.681959
107783773	LOC107783773	uncharacterized	brown	1395.553152
107805090	LOC107805090	probable methyltransferase PMT28	brown	1392.736808
novel.625	–	–	brown	1384.953429
107814737	LOC107814737	putative lipase YOR059C	brown	1382.143432
107828990	LOC107828990	uncharacterized	brown	1376.613385
107818553	LOC107818553	stomatin-like protein 2	green	964.7764105
107808848	LOC107808848	prohibitin-3	green	961.6056828
107800968	LOC107800968	prohibitin-1	green	961.2519135
107775921	LOC107775921	heat shock 70 kDa protein	green	957.9938316
107821521	LOC107821521	heat shock protein 90-6	green	955.9677757
107801899	LOC107801899	prohibitin-3	green	947.6532032
107791289	LOC107791289	LETM1 and EF-hand domain-containing protein 1	green	945.2186045
107768908	LOC107768908	protein DETOXIFICATION 35-like	green	941.2186707
107800496	LOC107800496	heat shock 70 kDa protein 15-like	green	938.6077192
107769814	LOC107769814	heat shock protein 90-6	green	938.0499536

In order to better illustrate the connections between the hub genes in the three modules, all modules were visualized.

**Figure 9:**
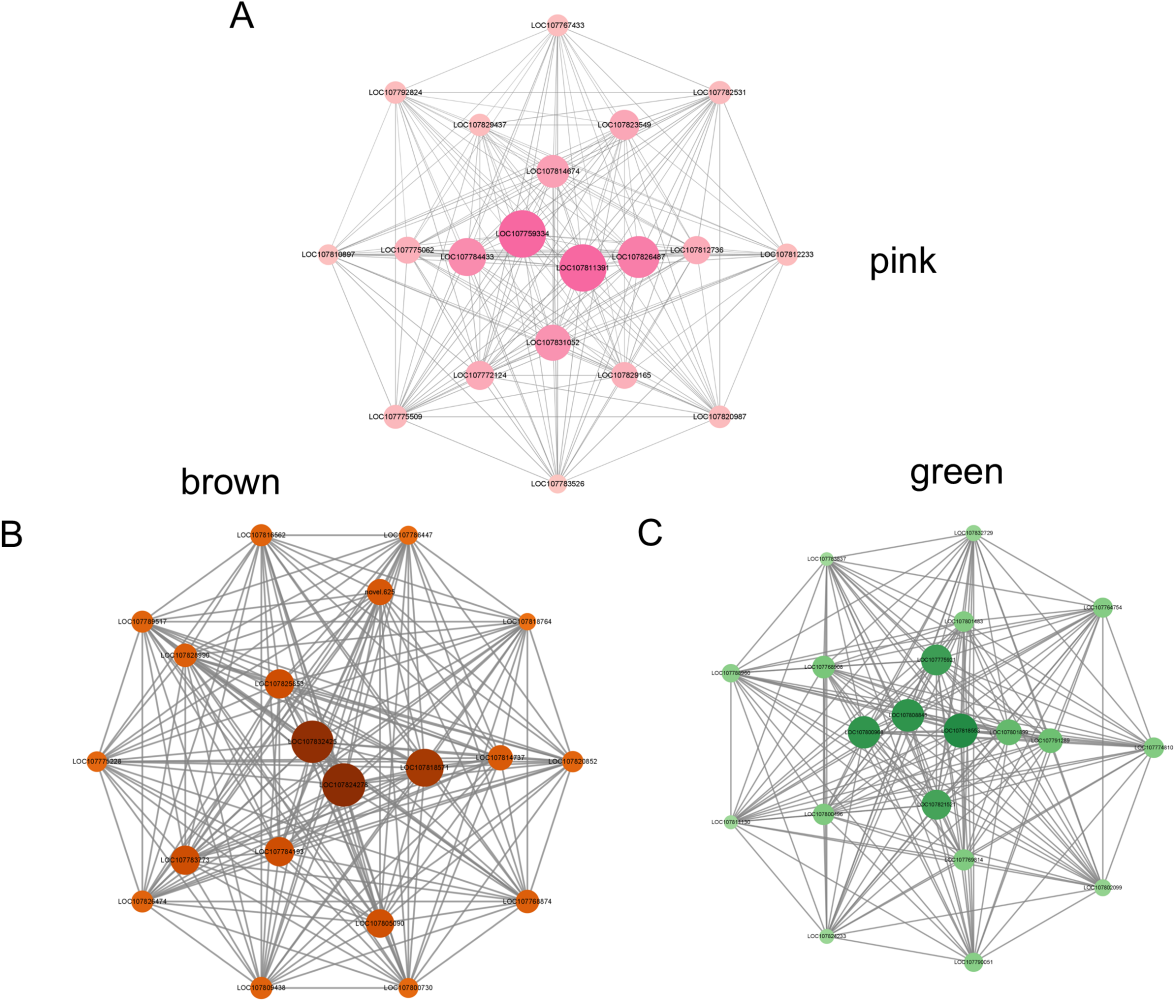
Gene connectivity graph of hub genes in the three modules. The darker the gene’s color and the larger its shape, the higher its k-Within. **(A)** pink module, **(B)** green module, **(C)** brown module.

The pink module was highly expressed in CV-1 only after 96h of low temperature treatment, which may be the reason why CV-1 was higher able to tolerate low temperature than K326. LOC107759334, LOC107811391, LOC107826487, LOC107784433, LOC107831052, LOC107814674, LOC107823549 and LOC107772124, LOC107812736, LOC107775062 were identified among the top ten k-Within in the pink module (**Figure 9A**). These genes are related to the synthesis and modification of carbohydrate proteins and lipids, cell membrane signal transduction and resistance to stress in tobacco cells. In response to low temperature stress, CV-1 regulates intracellular carbohydrate, protein and lipid synthesis and modification by receiving signal transduction from the cell membrane, and this difference in regulatory pathways may lead to the superior ability of CV-1 to cope with low temperature stress than K326.

The brown module was high expressed in both tobaccos before low temperature treatment and low expression after 96h. LOC107824278, LOC107832425, LOC107818571, LOC107784193, LOC107825853, LOC107783773, LOC107805090, novel. 625, LOC107814737 and LOC107828990 were identified in the top ten k-Within of the brown module (**Figure 9B**). However, LOC107783773 and LOC107828990 have not been characterized, and novel. 625 has not been identified. These genes are related to tobacco cell signal transduction, substance transport and energy metabolism. This indicates that substance transport and energy metabolism in tobacco are correspondingly weakened under low-temperature stress, which is also the main manifestation of damage to tobacco after low-temperature stress.

The green module, in contrast to the expression pattern of the brown module, was lowly expressed in both tobaccos before low temperature treatment and was high after 96h. LOC107818553, LOC107808848, LOC107800968, LOC107775921, LOC107821521, LOC107801899, LOC107791289, LOC107768908, LOC107800496 and LOC107769814 in the top ten k-Within of the green module (**Figure 9C**). These genes are primarily associated with the SPFH protein superfamily and heat shock proteins in tobacco. These proteins are key components of the plant’s response to environmental stress.

**Figure 10:**
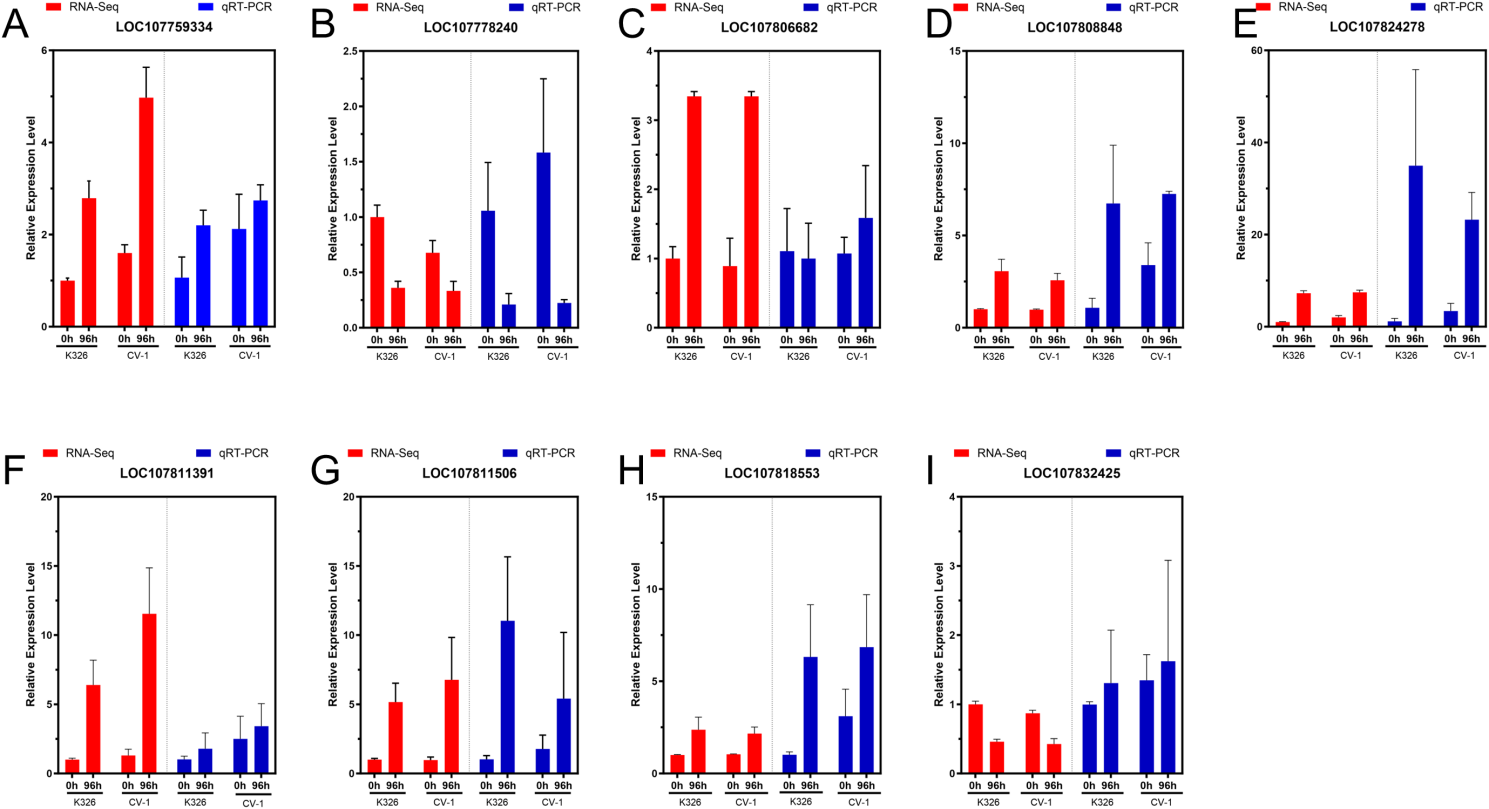
Comparison of qRT-PCR and RNA sequencing results. **(A)** LOC107759334, **(B)** LOC107778240, **(C)** LOC107806682, **(D)** LOC107808848, **(E)** LOC107824278, **(F)** LOC107811391, **(G)** LOC107811506, **(H)** LOC107818553, **(I)** LOC107832425.

To verify the accuracy of RNA sequencing results, we selected the top two hub genes in the three modules, and three differentially expressed genes were selected for qRT-PCR validation. The results of qRT-PCR were compared with those of RNA sequencing and found that the expression patterns of 7 of the 9 selected genes were consistent with those of RNA sequencing (**Figure 10**). The expression levels of LOC107759334 and LOC107811391 in the pink module, LOC107824278 in the brown module, and LOC107818553 and LOC107808848 in the green module, as well as LOC107811506 among the differentially expressed genes, were all downregulated after low-temperature treatment. However, the qRT-PCR trends of LOC107832425 in the brown module and LOC107806682 (K326) among the differentially expressed genes were different from those of RNA-Seq. This might be due to the fact that although we tried to maintain the same culture and treatment conditions for the samples used in qRT-PCR as those used in RNA-Seq, it is impossible to guarantee 100% reproducibility in RNA expression levels. Additionally, it cannot be ruled out that there were false positives in some samples during RNA-Seq.

## 4 Discussion

Plants face biotic stresses such as pests and diseases, as well as abiotic stresses such as drought and cold during their growth, which can significantly impair plant health and productivity. In order to cope with the stresses in the environment, plants need to undergo corresponding changes in morphology, biochemical metabolism and gene expression levels to adapt to the corresponding stresses.

In response to environmental stresses, ROS are produced in plants. A moderate amount of ROS acts as a signaling molecule to activate the expression of the corresponding genes. However, when the environmental stress is severe, excessive production of ROS will further aggravate the damage to cells. During the long evolutionary process, plants have evolved a complete set of intracellular mechanisms for scavenging ROS, among which three important antioxidant enzymes are CAT, POD and SOD. For the ROS produced in plants, SOD can decompose superoxide anion into hydrogen peroxide and molecular oxygen, while CAT and POD can further decompose hydrogen peroxide into water and molecular oxygen (Zhang et al., 2025). And ROS will cause peroxidation reaction of cell membrane, so that the structure and function of cell membrane receive damage. One of the typical products of cell membrane peroxidation is malondialdehyde, so MDA can represent the degree of damage to the cell membrane after stress (Li et al., 2025b).For the physiological and biochemical indexes of the two tobacco species, K326 and CV-1, it was shown that CV-1 had higher antioxidant enzyme activities and lower MDA content after cold stress, indicating that it was more resistant to low-temperature stress than K326.This is also why the CV-1 blades recover from wilting to a stiff state faster than the K326 blades in the middle and late stages of cold treatment.

To investigate the molecular mechanisms underlying the superior cold tolerance of K326 compared to CV-1, we selected leaves from K326 and CV-1 tobacco plants at 0 hours before low-temperature treatment and 96 hours after low-temperature treatment for transcriptomic sequencing. We used the NCBI reference genome ncbi_nicotiana_tabacum_gcf_000715135_1_ntab_tn90 as the reference genome for differential expression gene analysis and WGCNA analysis (Sierro et al., 2014).

Prior to low-temperature treatment, differentially expressed genes in EK vs EC group were enriched in cell movement-related pathways via GO analysis, while KEGG enrichment analysis indicated their association with motor proteins. This suggests significant differences in cell movement pathways between the two tobacco varieties, K326 and CV-1. Notably, DEGs in EK vs EC group were enriched in pathways related to phenylpropanoid synthesis. The phenylpropanoid metabolic pathway is one of the most important secondary metabolic pathways in plants and plays a crucial role in plant defense against biotic and abiotic stresses (Kumar et al., 2023). This suggests that CV-1 has a stronger ability to synthesize phenylpropanoids, which may be one of the reasons for its stronger resistance to low-temperature stress compared to K326.

After 96 hours of low-temperature treatment, the differentially expressed genes in EKA vs ECA group were enriched in GO pathways related to NADH synthesis and antioxidant enzymes, while KEGG enrichment revealed pathways related to nicotinic acid metabolism and peroxisomes. NADH is closely associated with cellular energy metabolism and antioxidant pathways. As an important carrier in the electron transport chain, NADH transfers high-energy electrons generated by metabolic activities to mitochondria. NADH also supports antioxidant defense by participating in glutathione reduction, helping cells resist oxidative stress (Hong et al., 2024). Peroxisomes contain abundant peroxidase, which effectively removes hydrogen peroxide produced by cells. CV-1 has stronger NADH synthesis capacity and antioxidant enzyme activities, thereby exhibiting stronger resistance to low-temperature stress. Notably, CV-1 exhibits enhanced copper ion binding pathways, and copper ions serve as essential cofactors for enzymes such as SOD and polyphenol oxidase (Zhou et al., 2022). This suggests that CV-1’s superior ability to withstand low-temperature stress may be attributed to its enhanced activity of SOD and other enzymes.

WGCNA analysis further revealed why CV-1 was able to adapt to the cold environment more quickly than K326 in the later stages of cold stress. We identified three modules that may contribute to the differences in cold resistance between the two groups. The pink module was specifically highly expressed only in CV-1, the green module was specifically highly expressed only after cold treatment, and the brown module was specifically highly expressed only before cold treatment.

The difference in the expression pattern of the central genes in the pink module may be the reason why CV-1 has stronger adaptive recovery ability than K326 from the second to the fourth day after low-temperature stress. SUMOylation plays an important role in plant response to environmental stress. The expression of *CaDeSI2* gene in pepper can affect the deSUMOylation process of *CaAITP1*, resulting in the reduction of its stability, thereby positively regulating the stress resistance of pepper (Joo et al., 2024). The expression pattern of *NtDeSI1L* may be similar to that of *CaDeSI2* and positively regulate stress tolerance in tobacco.*EDS1* is closely associated with plant immunity and can enhance plant defense against fungi through phosphorylation in Arabidopsis (Li et al., 2022). Transcriptomic analysis of tobacco suggests that *NtEDS1L* may be involved in responding to low-temperature stress and its phosphorylation may improve cold resistance in tobacco. Transgenic tobacco overexpressing *NtEXPA1* and *NtEXPA5* exhibited higher salt tolerance (Dabravolski and Isayenkov, 2025). With CV-1 gene expression levels higher than those in K326, it was consistent with their stronger adaptation to low-temperature stress than K326 during the late stages of cold stress, indicating that *NtEXPA2L* may be a positive regulator of cold tolerance. Compared to K326, CV-1 exhibits enhanced cold tolerance, likely due to its unique phosphorylation and sumoylation modifications of certain proteins such as *NtDeSI1L*, *NtEDS1L*, and *NtEXPA2L* in response to cold stress.

The brown module is negatively correlated in both K326 and CV-1 after 96 hours of low-temperature treatment. Plant pyruvate kinase is divided into cytosolic pyruvate kinase and plastid pyruvate kinase. *OsPK2* in rice chloroplasts can influence starch synthesis during rice development, while pyruvate kinase isozyme A is located in tobacco chloroplasts and may also play an important role in the conversion of photosynthetic products (Cai et al., 2018). The arm repeat protein interacting with *ABF2* is a key gene for isoflavone synthesis in soybean seeds. Isoflavones possess strong antioxidant capabilities and act as effective free radical scavengers, playing a crucial role in plants’ responses to biotic and abiotic stresses (Azam et al., 2023). The arm repeat protein interacting with *ABF2* may function as a negative regulator of isoflavone synthesis in tobacco. The *GN1.1* gene in rice can interact with the ADP-ribosylation factor GTPase-activating protein *OsZAC* and enhance its stability, affecting the growth of rice (Zhao et al., 2024). In tobacco, the expression of the *NtAGD6* protein is weakened after cold stress, potentially affecting auxin transport and weakening leaf growth and development. The FAM10 family protein *At4g22670* is an *Hsc70*-interacting protein in Arabidopsis. *Hsc70–1* acts as a negative regulator of *HsfA1d/A1e/A2* activators, thereby modulating *Hsp101* expression and basal heat tolerance. Overexpression of *Hsc70–1* leads to increased heat sensitivity in Arabidopsis (Tiwari et al., 2020). In tobacco, *NtHsc70* negatively regulates *At4g2267-like*. The expression of the *At4g2267* gene in CV-1 is reduced to a lesser extent, indicating that *Hsc70* expression levels are lower than in K326, thereby conferring higher resistance to cold stress. Research has found that in Arabidopsis thaliana, the water channel protein PIP can facilitate the transmembrane diffusion of hydrogen peroxide (Israel et al., 2022). In CV-1 cells, the expression level of the *pTOM75* gene decreases less than that of K326, indicating that it still has a high capacity to transport hydrogen peroxide to guard cells to respond to stress when coping with cold stress damage. When exposed to low-temperature stress, some physiological activities of CV-1 and K326 decrease. However, CV-1 still maintains high photosynthesis, isoflavone synthesis, and hydrogen peroxide transport, giving it higher cold tolerance than K326.

After 96 hours of low-temperature treatment, the brown modules showed a positive correlation in both K326 and CV-1.Stomatin-like protein plays a role in the organization of the respiratory supercomplex in Arabidopsis, affecting the activity of complex I and supercomplex I2III4 in the electron transport chain (Gehl et al., 2014). When its expression level is enhanced, the catalytic activity of the respiratory complex is also enhanced. *PHB* participates in the regulation of multiple hormone signaling pathways in plants. Arabidopsis and tomato *PHB3* interact with the brassinosteroid receptor *BRI1* and co-receptor *BAK1* to regulate the BR signaling pathway (Li et al., 2023a).*NtSLP2*, *NtPHB3*, and *NtPHB1* all belong to the SPFH protein superfamily and play important roles in plants’ responses to environmental stress. The increased expression of heat shock 70 kDa protein 1/6/8 in wheat enhances its resistance to cadmium and reduces cadmium accumulation (Wang et al., 2025). In ginger, *ZoHSP90.4* exhibits a unique dual response pattern under both heat stress and cold stress conditions. *ZoHSP90.4* reaches its peak expression after 6 hours of low-temperature stress and remains highly expressed 24 hours after high-temperature stress (Xiao et al., 2025). CV-1 expressed more *HSP90–6* and heat shock 70 kDa protein, which improved its cold resistance. Compared with K326, CV-1 exhibits higher levels of SPFH protein superfamily and heat shock protein family expression in response to low-temperature stress, thereby demonstrating higher low-temperature tolerance.

Compared to the cold-sensitive K326, the cold-tolerant tobacco CV-1 exhibits enhanced phenylpropanoid, isoflavone, and NADH synthesis capabilities. CV-1 also exhibits enhanced photosynthetic capacity and heat shock protein expression. These pathways have been previously identified in other cold-tolerant tobacco varieties. Cold-tolerant tobacco NC102 upregulates key genes and metabolites in the lignin biosynthesis pathway under low-temperature stress and demonstrates higher antioxidant enzyme activity (Li et al., 2025a). Cold-tolerant tobacco Xiangyan 7 also upregulates genes related to flavonoid and phenylpropanoid biosynthesis under cold stress (Hu et al., 2022). Following shallow-water sowing cultivation, Xiangyan 7 additionally upregulates genes associated with NADH synthesis and photosynthesis after cold treatment (Tao et al., 2024). Upregulation of heat shock protein-related genes was also identified in the cold-tolerant tobacco cultivar NC567 (Hu et al., 2016). Compared to K326, CV-1 exhibited unique phosphorylation and sumoylation of proteins such as DeSI1L, EDS1L, and EXPA2L, suggesting distinctive transcriptional responses to cold stress in the CV-1 cultivar.

## 5 Conclusion

**Figure 11:**
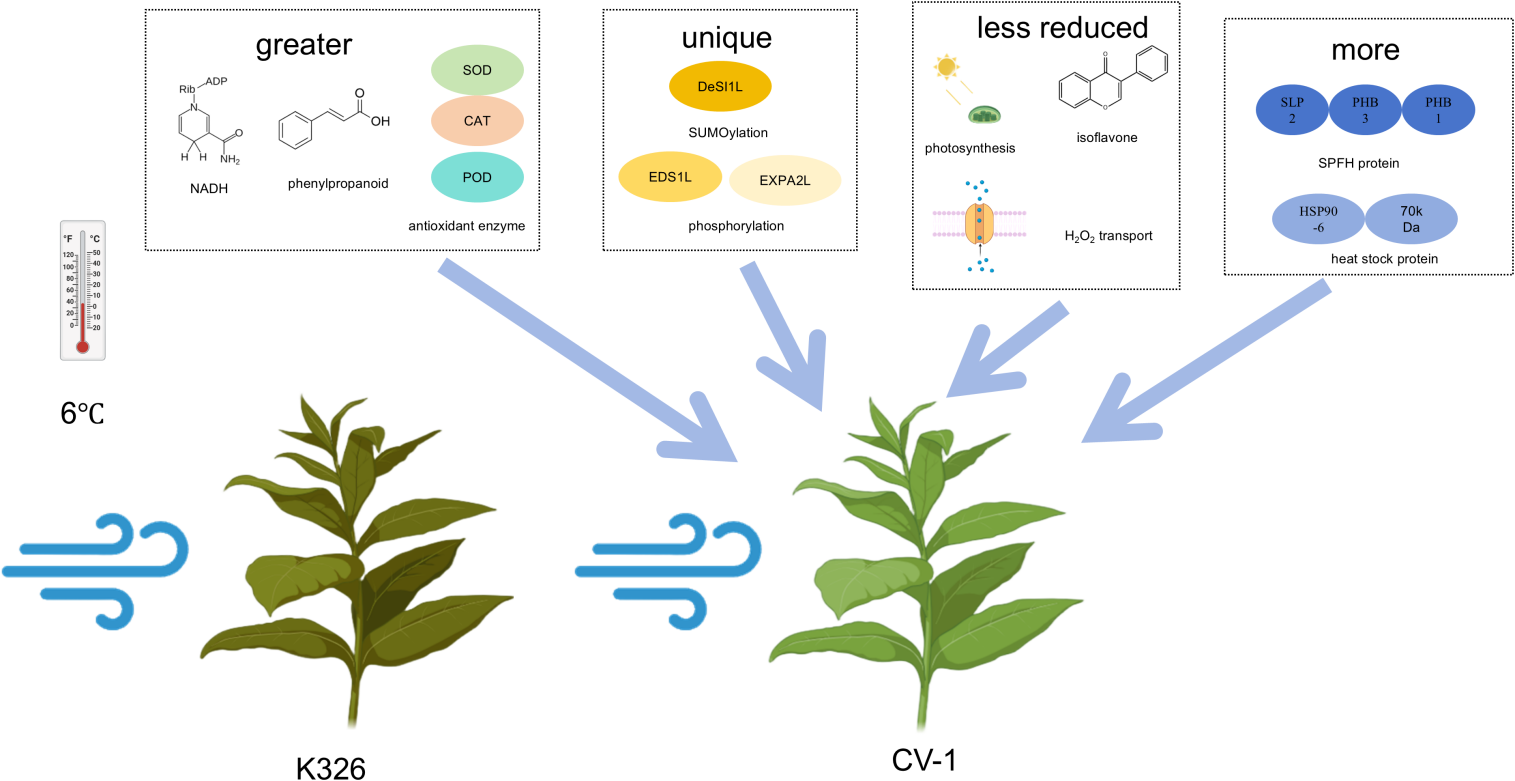
Mechanism of CV-1 being more cold-resistant than K326.

Through phenotypic, physiological and biochemical comparisons of K326 and CV-1, we found that CV-1 exhibits stronger cold tolerance. Upon exposure to cold stress, CV-1 demonstrates higher antioxidant enzyme activities and lower membrane damage levels. The greater cold tolerance of CV-1 may be due to its greater phenylpropanoid synthesis, NADH synthesis, and antioxidant enzyme system activities. In addition, its unique phosphorylation and SUMOylation of proteins such as DeSI1L, EDS1L, and EXPA2L, as well as stronger photosynthetic capacity, hydrogen peroxide transport capacity, isoflavone synthesis capacity, and high expression of the SPFH protein superfamily as well as the heat shock protein family may also confer stronger cold tolerance (**Figure 11**). Through transcriptomic comparison analysis, we identified some potential key genes responsible for the differing cold tolerance between the two tobacco varieties. In the next step, we will validate the specific mechanisms of these key genes through overexpression or gene knockout. In summary, this study provides valuable transcriptomic resources for investigating the cold adaptation mechanisms of tobacco varieties.

## Data Availability

The datasets presented in this study are publicly available. This data can be found here: https://www.ncbi.nlm.nih.gov/sra, accession number PRJNA1327801.

## References

[B1] Anders S. Huber W. (2010). Differential expression analysis for sequence count data. Genome Biol. 11, R106. doi: 10.1186/gb-2010-11-10-r106, PMID: 20979621 PMC3218662

[B2] Azam M. Zhang S. Huai Y. Abdelghany A. M. Shaibu A. S. Qi J. . (2023). Identification of genes for seed isoflavones based on bulk segregant analysis sequencing in soybean natural population. Theor. Appl. Genet. 136, 13. doi: 10.1007/s00122-023-04258-5, PMID: 36662254

[B3] Cai Y. Li S. Jiao G. Sheng Z. Wu Y. Shao G. . (2018). OsPK2 encodes a plastidic pyruvate kinase involved in rice endosperm starch synthesis, compound granule formation and grain filling. Plant Biotechnol. J. 16, 1878–1891. doi: 10.1111/pbi.12923, PMID: 29577566 PMC6181219

[B4] Dabravolski S. A. Isayenkov S. V. (2025). Expansins in salt and drought stress adaptation: from genome-wide identification to functional characterisation in crops. Plants 14, 1327. doi: 10.3390/plants14091327, PMID: 40364355 PMC12073716

[B5] Ding Y. L. Shi Y. T. Yang S. H. (2019). Advances and challenges in uncovering cold tolerance regulatory mechanisms in plants. New Phytol. 222, 1690–1704. doi: 10.1111/nph.15696, PMID: 30664232

[B6] Gehl B. Lee C. P. Bota P. Blatt M. R. Sweetlove L. J. (2014). An arabidopsis stomatin-like protein affects mitochondrial respiratory supercomplex organization. Plant Physiol. 164, 1389–1400. doi: 10.1104/pp.113.230383, PMID: 24424325 PMC3938628

[B7] Gilmour S. J. Sebolt A. M. Salazar M. P. Everard J. D. Thomashow M. F. (2000). Overexpression of the arabidopsis CBF3 transcriptional activator mimics multiple biochemical changes associated with cold acclimation1. Plant Physiol. 124, 1854–1865. doi: 10.1104/pp.124.4.1854, PMID: 11115899 PMC59880

[B8] Gu K. Y. Li X. K. Su J. E. Chen Y. Yang C. W. Li J. . (2024). Physiological and ecological responses of flue-cured tobacco to field chilling stress: insights from metabolomics and proteomics. Front. Plant Sci. 15. doi: 10.3389/fpls.2024.1490633, PMID: 39670264 PMC11635995

[B9] Gusain S. Joshi S. Joshi R. (2023). Sensing, signalling, and regulatory mechanism of cold-stress tolerance in plants. Plant Physiol. Biochem. 197, 107646. doi: 10.1016/j.plaphy.2023.107646, PMID: 36958153

[B10] He X. Liu T. X. Ren K. Chen J. Zhao G. K. Hu B. B. . (2020). Salicylic acid effects on flue-cured tobacco quality and curing characteristics during harvesting and curing in cold-stressed fields. Front. Plant Sci. 11. doi: 10.3389/fpls.2020.580597, PMID: 33193524 PMC7661750

[B11] Hong Y. Yu Z. Zhou Q. Chen C. Hao Y. Wang Z. . (2024). NAD^+^ deficiency primes defense metabolism *via*^1^O_2_-escalated jasmonate biosynthesis in plants. Nat. Commun. 15, 6652. doi: 10.1038/s41467-024-51114-1, PMID: 39103368 PMC11300881

[B12] Hu Z. Yan W. Yang C. Huang X. Hu X. Li Y. . (2022). Integrative analysis of transcriptome and metabolome provides insights into the underlying mechanism of cold stress response and recovery in two tobacco cultivars. Environ. Exp. Bot. 200, 104920. doi: 10.1016/j.envexpbot.2022.104920

[B13] Hu R. Zhu X. Xiang S. Zhan Y. Zhu M. Yin H. . (2016). Comparative transcriptome analysis revealed the genotype specific cold response mechanism in tobacco. Biochem. Biophys. Res. Commun. 469, 535–541. doi: 10.1016/j.bbrc.2015.12.040, PMID: 26692485

[B14] Huang L. Hong Y. Zhang H. Li D. Song F. (2016). Rice NAC transcription factor ONAC095 plays opposite roles in drought and cold stress tolerance. BMC Plant Biol. 16, 203. doi: 10.1186/s12870-016-0897-y, PMID: 27646344 PMC5029094

[B15] Israel D. Lee S. H. Robson T. M. Zwiazek J. J. (2022). Plasma membrane aquaporins of the PIP1 and PIP2 subfamilies facilitate hydrogen peroxide diffusion into plant roots. BMC Plant Biol. 22, 556. doi: 10.1186/s12870-022-03962-6, PMID: 36471241 PMC9721007

[B16] Jin J. J. Zhang H. Zhang J. F. Liu P. P. Chen X. Li Z. F. . (2017). Integrated transcriptomics and metabolomics analysis to characterize cold stress responses in *Nicotiana tabacum*. BMC Genomics 18, 15. doi: 10.1186/s12864-017-3871-7, PMID: 28662642 PMC5492280

[B17] Joo H. Baek W. Lim C. W. Lee S. C. (2024). Pepper SUMO protease CaDeSI2 positively modulates the drought responses *via* deSUMOylation of clade A PP2C CaAITP1. New Phytol. 243, 1361–1373. doi: 10.1111/nph.19920, PMID: 38934066

[B18] Kidokoro S. Shinozaki K. Yamaguchi-Shinozaki K. (2022). Transcriptional regulatory network of plant cold-stress responses. Trends Plant Sci. 27, 922–935. doi: 10.1016/j.tplants.2022.01.008, PMID: 35210165

[B19] Kumar A. P. Bhasker K. Nikhil B. S. K. Srinivas P. (2023). Role of phenylpropanoids and flavonoids in plant defense mechanism. Int. J. Environ. Climate Change 13, 2951–2960. doi: 10.9734/ijecc/2023/v13i92534

[B20] Langfelder P. Horvath S. (2008). WGCNA: an R package for weighted correlation network analysis. BMC Bioinf. 9, 559. doi: 10.1186/1471-2105-9-559, PMID: 19114008 PMC2631488

[B21] Li H. Dong S. Wang S. Song K. Zhang X. Yang L. (2025a). Transcriptomics and metabolomics reveal that seed cold priming enhances cold tolerance in tobacco seedlings by activating lignin biosynthesis and auxin signaling pathways. Ind. Crops Products. 236, 122034. doi: 10.1016/j.indcrop.2025.122034

[B22] Li J. Muneer M. A. Sun A. H. Guo Q. Y. Wang Y. M. Huang Z. R. . (2023b). Magnesium application improves the morphology, nutrients uptake, photosynthetic traits, and quality of tobacco (*Nicotiana tabacum* L.) under cold stress. Front. Plant Sci. 14. doi: 10.3389/fpls.2023.1078128, PMID: 36844047 PMC9948613

[B23] Li L. X. Niu Y. Z. Deng S. W. Zhu M. J. Wu X. F. Peng S. . (2025b). Metabolomics analysis to characterize the effects of flavonoids on tobacco seedlings under cold and hypoxia stress. J. Plant Interact. 20, 12. doi: 10.1080/17429145.2025.2474825

[B24] Li M. Wang X. H. Cao Y. Liu X. Lin Y. Ou Y. B. . (2013). Strength comparison between cold-inducible promoters of *Arabidopsis cor15a* and *cor15b* genes in potato and tobacco. Plant Physiol. Biochem. 71, 77–86. doi: 10.1016/j.plaphy.2013.06.021, PMID: 23886924

[B25] Li Y. Xue J. Wang F.-Z. Huang X. Gong B.-Q. Tao Y. . (2022). Plasma membrane-nucleo-cytoplasmic coordination of a receptor-like cytoplasmic kinase promotes EDS1-dependent plant immunity. Nat. Plants 8, 802–816. doi: 10.1038/s41477-022-01195-x, PMID: 35851623

[B26] Li C. Zhang S. Li J. Huang S. Zhao T. Lv S. . (2023a). PHB3 interacts with BRI1 and BAK1 to mediate brassinosteroid signal transduction in Arabidopsis and tomato. New Phytol. 241, 1510–1524. doi: 10.1111/nph.19469, PMID: 38130037

[B27] Luo Z. Zhou Z. Li Y. Tao S. Hu Z.-R. Yang J.-S. . (2022). Transcriptome-based gene regulatory network analyses of differential cold tolerance of two tobacco cultivars. BMC Plant Biol. 22, 369. doi: 10.1186/s12870-022-03767-7, PMID: 35879667 PMC9316383

[B28] Ma X. H. Xu J. Y. Han D. Huang W. X. Dang B. J. Jia W. . (2020). Combination of β-aminobutyric acid and Ca^2+^ Alleviates chilling stress in tobacco (*Nicotiana tabacum* L.). Front. Plant Sci. 11. doi: 10.3389/fpls.2020.00556, PMID: 32477386 PMC7237732

[B29] Montes C. Wang P. Liao C. Y. Nolan T. M. Song G. Y. Clark N. M. . (2022). Integration of multi-omics data reveals interplay between brassinosteroid and Target of Rapamycin Complex signaling in Arabidopsis. New Phytol. 236, 893–910. doi: 10.1111/nph.18404, PMID: 35892179 PMC9804314

[B30] Sierro N. Battey J. N. D. Ouadi S. Bakaher N. Bovet L. Willig A. . (2014). The tobacco genome sequence and its comparison with those of tomato and potato. Nat. Commun. 5, 9. doi: 10.1038/ncomms4833, PMID: 24807620 PMC4024737

[B31] Solier S. Müller S. Cañeque T. Versini A. Mansart A. Sindikubwabo F. . (2023). A druggable copper-signalling pathway that drives inflammation. Nature 617, 386–394. doi: 10.1038/s41586-023-06017-4, PMID: 37100912 PMC10131557

[B32] Suo W. L. Li L. X. Zheng Y. Y. Pan S. S. Niu Y. Z. Guan Y. J. (2024). Effect of seed priming with zinc, iron and selenium on the low temperature tolerance of *Nicotiana tabacum* L. during seed germination. Biochem. Biophys. Res. Commun. 735, 11. doi: 10.1016/j.bbrc.2024.150806, PMID: 39427379

[B33] Tao X. Yang L. Zhang M. Li Y. Xiao H. Yu L. . (2024). Shallow water seeding cultivation enhances cold tolerance in tobacco seedlings. BMC Plant Biol. 24, 698. doi: 10.1186/s12870-024-05422-9, PMID: 39044176 PMC11267769

[B34] Tiwari L. D. Khungar L. Grover A. (2020). AtHsc70–1 negatively regulates the basal heat tolerance in Arabidopsis thaliana through affecting the activity of HsfAs and Hsp101. Plant J. 103, 2069–2083. doi: 10.1111/tpj.14883, PMID: 32573848

[B35] Wang Q. Li Y. Wang Y. Zhang M. Lv Y. Zhang H. . (2025). Integrative proteomic and physiological analyses reveal differential responses between high- and low-Cd-accumulating wheat under Cd stress. Ecotoxicol. Environ. Saf. 302, 118662. doi: 10.1016/j.ecoenv.2025.118662, PMID: 40663949

[B36] Wei S. Q. Chen M. J. Wang F. Y. Tu Y. S. Xu Y. F. Fu L. B. . (2025). OsCaM1–1 is responsible for salt tolerance by regulating Na^+^/K^+^ Homoeostasis in rice. Plant Cell Environ. 48, 1393–1408. doi: 10.1111/pce.15212, PMID: 39445791

[B37] Wei M. Zhuang Y. Li H. Li P. Huo H. Shu D. . (2019). The cloning and characterization of hypersensitive to salt stress mutant, affected in quinolinate synthase, highlights the involvement of NAD in stress-induced accumulation of ABA and proline. Plant J. 102, 85–98. doi: 10.1111/tpj.14613, PMID: 31733117

[B38] Xiao D. Jiang Y. Wang Z. Li X. Li H. Tang S. . (2025). Genome-wide identification and expression analysis of the HSP90 gene family in relation to developmental and abiotic stress in ginger (Zingiber officinale roscoe). Plants 14, 1660. doi: 10.3390/plants14111660, PMID: 40508332 PMC12157278

[B39] Xiao N. Ma H. Z. Wang W. X. Sun Z. K. Li P. P. Xia T. (2024). Overexpression of *ZmSUS1* increased drought resistance of maize (*Zea mays* L.) by regulating sucrose metabolism and soluble sugar content. Planta 259, 14. doi: 10.1007/s00425-024-04336-y, PMID: 38277077

[B40] Xie R. R. Wu S. X. Huang W. L. Luo Y. X. Lin J. B. Cheng Y. Z. . (2025). Assessment of cold resistance in tobacco varieties using JIP-test parameters and seedling growth. Physiol. Plant. 177, 14. doi: 10.1111/ppl.70078, PMID: 39868639

[B41] Zhang Y. Li L. J. Dai H. F. Kong X. J. Rahman M. Zhang B. H. . (2025). Iron oxide nanoparticles (FeO-NPs) mitigate salt stress in peanut seedlings by enhancing photosynthesis, osmoregulation, and antioxidant activity. Plant Physiol. Biochem. 227, 13. doi: 10.1016/j.plaphy.2025.110206, PMID: 40614542

[B42] Zhang T. Mo J. Zhou K. Chang Y. Liu Z. (2018). Overexpression of Brassica campestris BcICE1 gene increases abiotic stress tolerance in tobacco. Plant Physiol. Biochem. 132, 515–523. doi: 10.1016/j.plaphy.2018.09.039, PMID: 30312954

[B43] Zhao H. Y. Shan J. X. Ye W. W. Dong N. Q. Kan Y. Yang Y. B. . (2024). A QTL GN1.1, encoding FT-L1, regulates grain number and yield by modulating polar auxin transport in rice. J. Integr. Plant Biol. 66, 2158–2174. doi: 10.1111/jipb.13749, PMID: 39083298

[B44] Zhao Q. Xiang X. H. Liu D. Yang A. G. Wang Y. Y. (2018). Tobacco transcription factor *NtbHLH123* confers tolerance to cold stress by regulating the *NtCBF* pathway and reactive oxygen species homeostasis. Front. Plant Sci. 9. doi: 10.3389/fpls.2018.00381, PMID: 29643858 PMC5882786

[B45] Zhou G. Liu C. Cheng Y. Ruan M. Ye Q. Wang R. . (2022). Molecular evolution and functional divergence of stress-responsive Cu/Zn superoxide dismutases in plants. Int. J. Mol. Sci. 23, 7082. doi: 10.3390/ijms23137082, PMID: 35806085 PMC9266695

